# Methodological considerations regarding propofol versus sevoflurane induction in patients with mitral stenosis

**DOI:** 10.1016/j.bjane.2026.844832

**Published:** 2026-09-21

**Authors:** Aleza Ejaz

**Affiliations:** Ayub Medical College, Abbottabad, Pakistan

Dear Editor,

We read with interest the randomized clinical trial by Sherif et al.^1^ “Effects of propofol versus sevoflurane induction on echocardiographic parameters in patients with mitral stenosis: a randomized clinical trial”. This study addresses an important question in cardiac anesthesia and provides useful peri-induction echocardiographic data. We would like to highlight three methodological considerations that may influence interpretation of the findings.

The potential influence of post-randomization co-interventions warrants attention. Baseline characteristics were similar, but patients receiving propofol required significantly more phenylephrine during induction than those receiving sevoflurane. Phenylephrine may alter systemic vascular resistance, ventricular loading conditions, and Doppler-derived indices, including transmitral pressure gradients, pressure half-time, and calculated mitral valve area. Because it was administered after randomization, this difference represents a post-randomization co-intervention that may have influenced the echocardiographic findings. The authors acknowledge the potential influence of concomitant vasopressors but do not discuss the implications of the between-group difference in phenylephrine exposure. Clarifying its timing and extent of use would help readers assess the extent to which the observed differences may reflect the anesthetic strategy or differences in peri-induction hemodynamic management.^2^

The sample-size calculation also merits clarification. It appears to have been based on detecting a within-group reduction in mean pressure gradient after induction, whereas the stated objective of the trial was a between-group comparison of two anesthetic strategies. These represent different statistical hypotheses and require different assumptions about the treatment effect and variance. A sample size sufficient for paired pre-induction and post-induction comparisons may not necessarily provide adequate power for a between-group superiority analysis. This distinction is especially relevant for outcomes such as mean pressure gradient, peak pressure gradient, and peak velocity, where nonsignificant between-group findings may reflect either a small treatment effect or limited statistical power.^3^

Baseline-adjusted analysis may also strengthen the interpretation of the primary echocardiographic outcomes. For randomized trials with continuous outcomes, analysis of covariance or an appropriate repeated-measures model can account for baseline measurements and provide a more direct estimate of the between-group treatment effect.^4^ The authors acknowledge that repeated-measures methods would have been preferable for serial hemodynamic variables. A similar consideration may apply to the echocardiographic endpoints that underpin the study's main conclusions. Such analyses could strengthen the interpretation of the reported differences in mitral valve area and pressure half-time by accounting for pre-induction measurements.

These methodological considerations suggest that the reported findings favoring sevoflurane should be interpreted with due caution, particularly because the outcomes were short-term echocardiographic measures that may be influenced by peri-induction hemodynamic management. Further studies using standardized peri-induction protocols and appropriate statistical methods are needed to confirm these findings.

## Data availability statement

No new data were created or analyzed in this article. Data sharing is not applicable to this article.

## Ethics approval

Not applicable.

## Informed consent

Not applicable.

## Declaration of AI and AI-assisted technologies

The author used ChatGPT (OpenAI) for language polishing and structural refinement only. The author reviewed and edited the output and takes full responsibility for the scientific content, interpretation, and conclusions of the manuscript.

## Author contributions

Aleza Ejaz: Conceptualization; methodology; literature review; writing-original draft; writing-review & editing.

## Funding

The author declares that no funds, grants, or other support were received during the preparation of this manuscript.

## Conflicts of interest

The author declares no conflicts of interest.
